# Supplementary material for: Modeling glioblastoma heterogeneity as a dynamic network of cell states
Source: Mol Syst Biol. 2021 Sep 16;17(9):e10105. doi: 10.15252/msb.202010105 (PMC8444284; doi:10.15252/msb.202010105)
Supplement: Supplementary file 5 — Source Data for Figure 3 [file MSB-17-e10105-s001.zip › Figure3A_sourcedata/GSEA_3065/nosigdb_state6.GseaPreranked.1622718443993/RICHARDS_NOWAKOWSKI_SCIENCE_2017_NIN3_UPREG.html]

Details for gene set RICHARDS\_NOWAKOWSKI\_SCIENCE\_2017\_NIN3\_UPREG[GSEA]

|  || Dataset | state6 |
| Phenotype | NoPhenotypeAvailable |
| Upregulated in class | na\_pos |
| GeneSet | RICHARDS\_NOWAKOWSKI\_SCIENCE\_2017\_NIN3\_UPREG |
| Enrichment Score (ES) | 0.3106273 |
| Normalized Enrichment Score (NES) | 1.3774767 |
| Nominal p-value | 0.016438356 |
| FDR q-value | 0.10806733 |
| FWER p-Value | 0.94 |
Table: GSEA Results Summary

  

Fig 1: Enrichment plot: RICHARDS\_NOWAKOWSKI\_SCIENCE\_2017\_NIN3\_UPREG      
 Profile of the Running ES Score & Positions of GeneSet Members on the Rank Ordered List

  

| PROBE | GENE SYMBOL | GENE\_TITLE | RANK IN GENE LIST | RANK METRIC SCORE | RUNNING ES | CORE ENRICHMENT || 1 | SOX4 |  |  | 3 | 0.375 | 0.0366 | Yes |
| 2 | SOX11 |  |  | 70 | 0.230 | 0.0516 | Yes |
| 3 | SLC25A6 |  |  | 96 | 0.216 | 0.0699 | Yes |
| 4 | PNRC1 |  |  | 123 | 0.202 | 0.0868 | Yes |
| 5 | ASAP1 |  |  | 141 | 0.196 | 0.1041 | Yes |
| 6 | TET2 |  |  | 150 | 0.192 | 0.1220 | Yes |
| 7 | COX7A2L |  |  | 273 | 0.157 | 0.1234 | Yes |
| 8 | RAP2B |  |  | 280 | 0.154 | 0.1379 | Yes |
| 9 | FGFR1OP2 |  |  | 293 | 0.152 | 0.1515 | Yes |
| 10 | TP53INP1 |  |  | 342 | 0.145 | 0.1602 | Yes |
| 11 | CITED2 |  |  | 360 | 0.142 | 0.1722 | Yes |
| 12 | HNRNPH1 |  |  | 378 | 0.139 | 0.1839 | Yes |
| 13 | PDE4DIP |  |  | 392 | 0.137 | 0.1959 | Yes |
| 14 | EIF1 |  |  | 406 | 0.135 | 0.2077 | Yes |
| 15 | DPYSL3 |  |  | 464 | 0.127 | 0.2136 | Yes |
| 16 | DNAJB6 |  |  | 466 | 0.127 | 0.2260 | Yes |
| 17 | FBXW7 |  |  | 498 | 0.124 | 0.2346 | Yes |
| 18 | PAFAH1B3 |  |  | 536 | 0.120 | 0.2420 | Yes |
| 19 | RPL21 |  |  | 554 | 0.118 | 0.2516 | Yes |
| 20 | SATB1 |  |  | 566 | 0.117 | 0.2618 | Yes |
| 21 | NEDD4L |  |  | 615 | 0.113 | 0.2674 | Yes |
| 22 | SH3BGRL3 |  |  | 700 | 0.106 | 0.2680 | Yes |
| 23 | ZC3H14 |  |  | 717 | 0.104 | 0.2765 | Yes |
| 24 | GFPT1 |  |  | 773 | 0.100 | 0.2800 | Yes |
| 25 | RBP1 |  |  | 788 | 0.099 | 0.2881 | Yes |
| 26 | HSPA1B |  |  | 814 | 0.097 | 0.2948 | Yes |
| 27 | TPGS2 |  |  | 913 | 0.090 | 0.2923 | Yes |
| 28 | PFN2 |  |  | 942 | 0.089 | 0.2978 | Yes |
| 29 | PDHA1 |  |  | 977 | 0.087 | 0.3024 | Yes |
| 30 | BROX |  |  | 981 | 0.087 | 0.3106 | Yes |
| 31 | SCP2 |  |  | 1067 | 0.082 | 0.3089 | No |
| 32 | RND3 |  |  | 1213 | 0.075 | 0.2995 | No |
| 33 | TCF4 |  |  | 1234 | 0.074 | 0.3045 | No |
| 34 | SLAIN1 |  |  | 1325 | 0.070 | 0.3010 | No |
| 35 | CLK1 |  |  | 1366 | 0.069 | 0.3031 | No |
| 36 | KLF6 |  |  | 1424 | 0.067 | 0.3031 | No |
| 37 | LSM14A |  |  | 1466 | 0.066 | 0.3048 | No |
| 38 | PPP4R2 |  |  | 1519 | 0.063 | 0.3050 | No |
| 39 | KALRN |  |  | 1543 | 0.062 | 0.3085 | No |
| 40 | DIP2C |  |  | 1746 | 0.055 | 0.2906 | No |
| 41 | CDKN1C |  |  | 1788 | 0.054 | 0.2911 | No |
| 42 | HSPE1 |  |  | 1803 | 0.054 | 0.2948 | No |
| 43 | RPS6KB1 |  |  | 1853 | 0.052 | 0.2942 | No |
| 44 | HIP1 |  |  | 1868 | 0.052 | 0.2977 | No |
| 45 | CHD7 |  |  | 1953 | 0.049 | 0.2928 | No |
| 46 | CCT4 |  |  | 1969 | 0.049 | 0.2959 | No |
| 47 | STIP1 |  |  | 2150 | 0.043 | 0.2793 | No |
| 48 | DNMT3A |  |  | 2392 | 0.036 | 0.2551 | No |
| 49 | DIMT1 |  |  | 2461 | 0.035 | 0.2506 | No |
| 50 | MNAT1 |  |  | 2512 | 0.033 | 0.2481 | No |
| 51 | KLHL13 |  |  | 2530 | 0.033 | 0.2493 | No |
| 52 | FIGN |  |  | 2587 | 0.031 | 0.2459 | No |
| 53 | PRPF40A |  |  | 2811 | 0.026 | 0.2227 | No |
| 54 | MAP1B |  |  | 2893 | 0.024 | 0.2158 | No |
| 55 | UQCRC2 |  |  | 2924 | 0.024 | 0.2146 | No |
| 56 | DCLK2 |  |  | 3031 | 0.021 | 0.2045 | No |
| 57 | ACTR10 |  |  | 3069 | 0.021 | 0.2022 | No |
| 58 | DDX3Y |  |  | 3174 | 0.018 | 0.1920 | No |
| 59 | MTRNR2L1 |  |  | 3298 | 0.015 | 0.1793 | No |
| 60 | SFSWAP |  |  | 3303 | 0.015 | 0.1804 | No |
| 61 | USP9Y |  |  | 3396 | 0.013 | 0.1711 | No |
| 62 | KRAS |  |  | 3447 | 0.012 | 0.1665 | No |
| 63 | POLE3 |  |  | 3486 | 0.012 | 0.1632 | No |
| 64 | SMARCA5 |  |  | 3558 | 0.010 | 0.1560 | No |
| 65 | HSPB1 |  |  | 3587 | 0.010 | 0.1537 | No |
| 66 | ST7 |  |  | 3606 | 0.009 | 0.1525 | No |
| 67 | WDR44 |  |  | 3656 | 0.008 | 0.1476 | No |
| 68 | PLXNA4 |  |  | 3824 | 0.004 | 0.1288 | No |
| 69 | NDUFAB1 |  |  | 3836 | 0.004 | 0.1279 | No |
| 70 | IGF1R |  |  | 3930 | 0.002 | 0.1173 | No |
| 71 | NUDCD2 |  |  | 3934 | 0.002 | 0.1171 | No |
| 72 | RAB5B |  |  | 3937 | 0.002 | 0.1171 | No |
| 73 | GABBR1 |  |  | 4201 | -0.003 | 0.0870 | No |
| 74 | UTP23 |  |  | 4293 | -0.005 | 0.0770 | No |
| 75 | TIAM1 |  |  | 4472 | -0.008 | 0.0572 | No |
| 76 | DNAJB1 |  |  | 4510 | -0.009 | 0.0538 | No |
| 77 | SLC25A36 |  |  | 4534 | -0.009 | 0.0521 | No |
| 78 | FOS |  |  | 4536 | -0.010 | 0.0529 | No |
| 79 | RASA1 |  |  | 4620 | -0.011 | 0.0444 | No |
| 80 | FOXRED1 |  |  | 4636 | -0.011 | 0.0438 | No |
| 81 | MARK3 |  |  | 4659 | -0.012 | 0.0424 | No |
| 82 | BRINP2 |  |  | 4802 | -0.014 | 0.0274 | No |
| 83 | GAD1 |  |  | 4858 | -0.016 | 0.0226 | No |
| 84 | ASF1A |  |  | 4888 | -0.016 | 0.0209 | No |
| 85 | TRAPPC2 |  |  | 4976 | -0.018 | 0.0126 | No |
| 86 | RBM4B |  |  | 5095 | -0.020 | 0.0009 | No |
| 87 | ADAR |  |  | 5098 | -0.020 | 0.0027 | No |
| 88 | CNOT2 |  |  | 5208 | -0.022 | -0.0077 | No |
| 89 | SACS |  |  | 5232 | -0.023 | -0.0081 | No |
| 90 | EPHA4 |  |  | 5330 | -0.025 | -0.0169 | No |
| 91 | PLS3 |  |  | 5389 | -0.026 | -0.0210 | No |
| 92 | LYRM2 |  |  | 5442 | -0.027 | -0.0243 | No |
| 93 | ACTB |  |  | 5477 | -0.028 | -0.0255 | No |
| 94 | GPBP1 |  |  | 5513 | -0.029 | -0.0267 | No |
| 95 | LSAMP |  |  | 5577 | -0.030 | -0.0310 | No |
| 96 | FAM193B |  |  | 5608 | -0.031 | -0.0315 | No |
| 97 | HSF2 |  |  | 5638 | -0.031 | -0.0317 | No |
| 98 | SERINC5 |  |  | 5716 | -0.033 | -0.0374 | No |
| 99 | CBL |  |  | 6083 | -0.040 | -0.0757 | No |
| 100 | CCDC88A |  |  | 6086 | -0.040 | -0.0720 | No |
| 101 | MFN1 |  |  | 6092 | -0.040 | -0.0687 | No |
| 102 | ARPC3 |  |  | 6141 | -0.041 | -0.0702 | No |
| 103 | TTTY15 |  |  | 6142 | -0.041 | -0.0661 | No |
| 104 | C2orf68 |  |  | 6217 | -0.043 | -0.0704 | No |
| 105 | CHORDC1 |  |  | 6278 | -0.044 | -0.0730 | No |
| 106 | HOOK3 |  |  | 6406 | -0.047 | -0.0830 | No |
| 107 | CLOCK |  |  | 6417 | -0.048 | -0.0795 | No |
| 108 | MAPK10 |  |  | 6491 | -0.049 | -0.0831 | No |
| 109 | HSPH1 |  |  | 6617 | -0.053 | -0.0923 | No |
| 110 | HSP90AB1 |  |  | 6619 | -0.053 | -0.0871 | No |
| 111 | SETX |  |  | 6774 | -0.058 | -0.0992 | No |
| 112 | MYL6 |  |  | 6811 | -0.059 | -0.0976 | No |
| 113 | HMBOX1 |  |  | 7045 | -0.066 | -0.1179 | No |
| 114 | RHOB |  |  | 7306 | -0.075 | -0.1406 | No |
| 115 | PKD2 |  |  | 7365 | -0.077 | -0.1398 | No |
| 116 | CENPC |  |  | 7474 | -0.080 | -0.1444 | No |
| 117 | CLK4 |  |  | 7477 | -0.080 | -0.1367 | No |
| 118 | SYNE2 |  |  | 7598 | -0.086 | -0.1421 | No |
| 119 | SOX6 |  |  | 7616 | -0.087 | -0.1355 | No |
| 120 | SUPT20H |  |  | 7691 | -0.091 | -0.1351 | No |
| 121 | ZNF738 |  |  | 7716 | -0.092 | -0.1289 | No |
| 122 | KIZ |  |  | 7779 | -0.094 | -0.1268 | No |
| 123 | TMEM123 |  |  | 7786 | -0.095 | -0.1182 | No |
| 124 | PPFIA1 |  |  | 7796 | -0.095 | -0.1098 | No |
| 125 | MUM1 |  |  | 7817 | -0.097 | -0.1026 | No |
| 126 | SGCB |  |  | 7845 | -0.098 | -0.0961 | No |
| 127 | ST8SIA5 |  |  | 7931 | -0.103 | -0.0958 | No |
| 128 | CACYBP |  |  | 8012 | -0.108 | -0.0944 | No |
| 129 | AMFR |  |  | 8037 | -0.109 | -0.0864 | No |
| 130 | BMPR2 |  |  | 8138 | -0.117 | -0.0865 | No |
| 131 | HSP90AA1 |  |  | 8153 | -0.117 | -0.0766 | No |
| 132 | UGGT2 |  |  | 8400 | -0.146 | -0.0906 | No |
| 133 | DST |  |  | 8644 | -0.211 | -0.0979 | No |
| 134 | SNTG1 |  |  | 8713 | -0.251 | -0.0811 | No |
| 135 | LAPTM4A |  |  | 8730 | -0.267 | -0.0566 | No |
| 136 | KCNQ1OT1 |  |  | 8742 | -0.281 | -0.0303 | No |
| 137 | MEG3 |  |  | 8786 | -0.370 | 0.0012 | No |
Table: GSEA details [plain text format]

  

Fig 2: RICHARDS\_NOWAKOWSKI\_SCIENCE\_2017\_NIN3\_UPREG: Random ES distribution      
 Gene set null distribution of ES for **RICHARDS\_NOWAKOWSKI\_SCIENCE\_2017\_NIN3\_UPREG**
